# Knowledge, Attitudes, and Practices Toward Antibiotic Use in Food-Producing Animals Among University Students in Seven Cities in Southern and Central China: A Cross-Sectional Study

**DOI:** 10.3390/antibiotics13121189

**Published:** 2024-12-06

**Authors:** Hui Sun, Jiajia Zhang, Junjie Zhu, Boya Xu, Yinyan Gao, Dexing Zhang, Irene X. Y. Wu, Yanhong Jessika Hu, Shuzhen Deng

**Affiliations:** 1 Baoshan Center for Disease Control and Prevention, Shanghai 200000, China; 2 Xiangya School of Public Health, Central South University, Changsha 410000, China; 3 Department of Epidemiology and Health Statistics, School of Public Health, Dali University, Dali 671000, China; 4 The Jockey Club School of Public Health and Primary Care, The Chinese University of Hong Kong, Hong Kong 999077, China; 5 Hunan Provincial Key Laboratory of Clinical Epidemiology, Changsha 410000, China; 6 Murdoch Children’s Research Institute, Royal Children’s Hospital, Parkville, VIC 3052, Australia; 7 Department of Paediatrics, The University of Melbourne, Parkville, VIC 3052, Australia; 8 Yunnan Center for Disease Control and Prevention, Kunming 650000, China

**Keywords:** knowledge, attitude, practice, food-producing animal, antibiotic

## Abstract

**Background:** The misuse of antibiotics in both humans and food-producing animals poses significant risks to human health and contributes to the rise of antibiotic resistance. Raising public awareness is crucial to managing antibiotic resistance, particularly among university students, as they represent a future force in tackling this global issue. **Methods:** A cross-sectional study was conducted from July 2022 to May 2024 in seven cities in Southern and Central China to assess university students’ knowledge, attitude, and practice regarding antibiotic use in humans and food-producing animals. Binary logistic regression was used to identify associated factors. **Results:** A total of 6357 students from 72 universities participated. Less than half of the students answered the knowledge items appropriately. Only 21.47% to 29.98% had a proper understanding of basic antibiotic concepts and their use in humans and food-producing animals. Respectively, 21.49% and 28.50% students paid attention to antibiotic content in food from food-producing animals and refused to buy food containing antibiotics. Factors associated with higher knowledge, attitude, and practice total scores included being male, being of older age, having a postgraduate education, majoring in the medical science discipline, studying at a double-first-class university, having a higher family monthly income, having parents in the medical area, and using antibiotics in the past year (*p* < 0.001). **Conclusions:** Given students’ insufficient knowledge—particularly in identifying antibiotics and understanding their functions—and inappropriate practices related to purchasing food from food-producing animals, targeted education programs are suggested. These programs should address the fundamental concepts of antibiotic use in both humans and food-producing animals while providing practical guidance on individual behaviors to help mitigate antibiotic resistance.

## 1. Introduction

Inappropriate antibiotic use across the human, animal, and environmental sectors is a major driver of antimicrobial resistance (AMR) [1,2]. Global data indicate that antibiotic consumption in the animal sector surpasses that in the human sector [3]. These antibiotics are commonly administered in food-producing animals for disease treatment and prevention [4]. However, while the non-medical use of antibiotics, such as for promotion of growth, has been prohibited in European countries for many years [5], such practices remain largely unregulated in developing nations [6]. The global usage of antimicrobials in food animals was estimated at 99,502 tons in 2020 based on data from 42 countries and was projected to increase by 8.0% to 107,472 tons by 2030 [7]. Asia accounted for 67% of the hotspots for antimicrobial use intensity, with many located in developing countries, including in Eastern China, Southern India, Indonesia, and Central Thailand [7]. Antibiotic use in food-producing animals can lead to the emergence of antibiotic-resistant bacterial, which may be transmitted to human through animal products, such as meat, eggs, and milk, and the accumulation of antibiotic residues, which can cause significant health issues, including allergic reactions, immunopathological effects, and even anaphylactic shock [8,9].

Given the profound and widespread risks associated with antibiotic use in food-producing animals, the World Health Organization (WHO) has issued guidelines advocating for the prudent use of antimicrobials in the industry, aiming to mitigate the acceleration of AMR [10]. Public awareness and education are crucial for the effective management and control of AMR [11]. As emphasized by the WHO, any intervention will be ineffective unless inappropriate antibiotic use behaviors are addressed [12]. Knowledge, attitude, and practice (KAP) studies are essential for identifying gaps in understanding and behaviors within specific social groups, providing the foundation for targeted interventions [12]. University students, with their higher educational experiences, can significantly influence public attitudes regarding antibiotic use. Addressing the challenge of AMR requires joint efforts across various sectors, including clinical medicine, public health, pharmacy, agriculture, livestock, and aquaculture. As future leaders and decision-makers in these fields, university students are poised to play a key role. Therefore, understanding their KAP towards antibiotic use in food-producing animals is crucial. Insights can inform actionable and targeted interventions or training, contributing to more effective management of AMR.

Several studies have explored university students’ KAP towards antibiotic use in food-producing animals; however, most have focused solely on students majoring in veterinary medicine or animal health. In a study by Dyar et al., which included 255 human and animal health students from 25 universities, only one-fifth of students felt they had sufficient knowledge on antibiotic use and less than half (44%) agreed with the statement “Animals can become resistant to antibiotics” [13]. In China, studies have also highlighted poor KAP among university students regarding antibiotic use, but few addressed questions specific to food-producing animals. One Chinese study on human antibiotics found that students with lower KAP scores were more likely to self-medicate with antibiotics or demand antibiotics from doctors [14]. To address the gap, a cross-sectional study was conducted across universities in seven cities within Yunnan, Hu’nan, and Henan Provinces, located in Southern and Central China. This study aimed to assess university students’ KAP regarding antibiotic use in food-producing animals and to identify potential influencing factors.

## 2. Results

### 2.1. Basic Information of Participants

A total of 7170 questionnaires were collected, of which 813 were excluded due to either short completion time or inconsistencies in the basic information provided. Finally, 6357 (88.66%) participants were included in the analysis. Females (n = 4645) accounted for 73.07% of the participants (Table 1). The mean age was 20.00 years (19.00–21.00 years). The participants were mainly undergraduates (n = 5898, 92.78%) and majoring in medical science (n = 3790, 59.62%). The parents of most participants (n = 4980, 78.34%) were non-medical staff. Nearly half of participants (n = 2906, 45.71%) reported a family monthly income between 3001 and 10,000 CNY. In terms of vaccination, 4428 (69.66%) participants received a seasonal influenza vaccine and 6275 (98.71%) had been vaccinated against COVID-19. Additionally, 4095 (64.42%) reported using antibiotics within the past year.

### 2.2. Appropriate Respondence of KAP Items

None of the knowledge items had an appropriateness rate above 50%. Nearly half of the participants appropriately answered the two knowledge items “Antibiotics are only effective for treating viral infections” (49.27%) and “Bacteria which are resistant to antibiotics can be spread from food-producing animal to human” (47.60%) (Table 2). The remaining six items had even lower appropriateness rates, especially those two items related to basic practice principles of antibiotics use in humans and food-producing animals. Only 21.47% of participants recognized that antibiotics are ineffective in treating the common cold and 22.34% believed that most local food-producing animals contain antibiotics.

All attitude items received moderate appropriateness rates. Respectively, 82.07% supported reducing antibiotic use and 38.37% of participants disagreed that antibiotics could be used as growth promoters in food-producing animals.

Regarding three practice items, 50.57% of the participants were willing to pay a higher price for antibiotic-free food from food-producing animals. However, only 21.49% frequently checked the antibiotic content when purchasing food from food-producing animals and 28.50% reported refusing to buy products due to the presence of antibiotics.

### 2.3. Factors Associated with KAP Scores

Several factors were positively associated with higher KAP total score, including being male, being of older age, having a postgraduate education, majoring in the medical science discipline, studying at a double-first-class university, having a higher family monthly income, having parents in the medical area, being vaccinated in the past year, and using antibiotics in the past year (Table 3). 

Older age, majoring in medical science, attending a double-first-class university, having parents in a medical area, and using antibiotics in the past year were common factors associated with scores of each KAP dimension (*p* < 0.05) (Table 4). Students of the medical science discipline demonstrated higher knowledge and attitude scores and lower practice scores (*p* < 0.05).

### 2.4. Correlation Analysis Between KAP

The total score showed a positive association with scores of each KAP dimension (*p* < 0.001). Spearman correlation coefficients were 0.120 between knowledge and attitude scores, 0.065 between knowledge and practice scores, and 0.082 between attitude and practice scores (Table 5).

## 3. Discussion

This study assessed 6357 students from seven cities in Southern and Central China regarding their KAP towards antibiotic use in food-producing animals. Our findings revealed that university students generally lacked sufficient knowledge about antibiotic identification, principles of use, and their application in food from food-producing animals. While students exhibited generally acceptable attitudes, they paid limited attention to antibiotic use in food from food-producing animals and were not proactive in their purchasing behaviors. Factors such as being male, being older, having a postgraduate education, being a medical science major, attending a double-first-class university, having a higher family monthly income, having parents in the medical field, and using antibiotics in the past year were significantly associated with higher KAP total score. Additionally, positive correlations were found between scores of each KAP dimension, as well as the KAP total score.

Despite acceptable attitudes, there was still a concerning gap. Attention should be paid to the item regarding attitude towards antibiotics used as growth promoters in food-producing animals, with slightly lower appropriate respondence rates of 38.37%. According to WHO guidelines, antibiotics should be restricted for growth promotion and disease prevention and their use in disease treatment should be limited to veterinary recommendations [15]. This finding underscored the need for targeted health education programs.

Knowledge and practice items revealed particularly low appropriateness rates. Only 21.47% to 29.98% of university students understood basic antibiotic concepts appropriately and their applications in humans. Additionally, just 22.34% and 24.41% were aware that local and imported food-producing animals often contain antibiotics. These results align with prior studies. For example, a survey of 1200 university students in the United Arab Emirates showed similarly low KAP scores regarding antibiotic use and limited knowledge regarding the identification of antibiotics and definition of antibiotic-related clinical testing [16]. A national study of veterinary students in Nigeria also revealed poor knowledge of antibiotic use, with over 60% scoring below average and 87% calling for more education on clinical antibiotic use and links between human, animal, and environmental health [17].

Regarding practice, only 21.49% and 28.50% of students respectively checked for antibiotic content in food from food-producing animals and refused to purchase food containing antibiotics. This could be due to students’ limited experience in purchasing food from food-producing animals. However, these low rates suggest a lack of awareness and attention to antibiotic use in food-producing animals. Despite existing measures, antibiotics remain commonly used in livestock [18]. Studies showed that antibiotic residues in animal-derived food, including meat, eggs, and milk in China, often exceeded safety limits, especially in fish [19]. Medical professionals have been advocating for recommending antibiotic-free food to patients, and university students, who are future leaders in society, must also adopt these behaviors early for their own and public health benefit [18]. 

The regression analysis revealed that university students who were male, older, postgraduates, medical science majors, from double-first-class universities, had higher family monthly income, had medical parents, or had experience using antibiotics in the past year scored higher in KAP. These findings are consistent with previous studies on antibiotic use in humans [16,20,21,22,23,24,25]. Compared to females, males exhibited higher KAP total scores towards antibiotic use in food-producing animals, possibly due to a significantly higher proportion of male postgraduates (6.72% versus 8.59%, *p* = 0.012) and males from double-first-class universities (9.26% versus 13.61%, *p* < 0.001), both of which are associated with enhanced access to advanced education and resources. Additionally, a significantly higher percentage of males reported having parents in the medical field (25.53% versus 20.24%, *p* < 0.001) compared to females, potentially fostering greater awareness and knowledge about antibiotics through familial influence.

Conversely, younger students from non-double-first-class universities and those without recent antibiotics use scored lower in each dimension, identifying that they, particularly those in their first year of university, should be a future target population for educational interventions. This approach can help instill good practices early on and reinforce the importance of antibiotic awareness and resistance prevention. Additionally, individuals who recently received influenza vaccinations had higher KAP total scores and scores for each dimension compared to those who were never vaccinated. This might be that some people were vaccinated due to positive perceptions of vaccination, which are correlated with appropriate knowledge and understanding of antibiotics, as suggested by a previous study [26]. Moreover, vaccines may reduce antibiotic use by preventing infections, with the effects of influenza vaccines being particularly evident [27,28]. The association is likely due to exposure to healthcare professionals and public health messaging during vaccination campaigns. However, the observed effect weakened over time, indicating the need for continuous education to maintain antibiotic stewardship. Integrating antibiotic-related messaging into vaccination programs could further boost long-term KAP scores, creating a well-rounded strategy to tackle antibiotic resistance. 

Although positive correlations were observed between scores of each KAP dimension, the correlation between knowledge and practice and between attitude and practice were relatively weak, with coefficients respectively of 0.065 and 0.082. Similar findings were reported in a cross-sectional study among Colombian medical students [20], where 28.5% recognized antibiotic resistance as a multifactorial issue but did not act accordingly, believing their individual actions had little impact [20]. The gap between knowledge and practice has been observed in several other studies [29,30]. To address this, educational programs should focus on practical actions that individuals can take to combat antibiotic resistance and emphasize the real-world impact of these behaviors. Incorporating hands-on activities in these programs is highly recommended. Additionally, students majoring in medical science demonstrated higher knowledge scores and attitude scores but lower practice scores compared to non-medical science majors. This finding highlights the need for targeted education programs that consider discipline and factors with contrasting associations, such as education and vaccination history, to improve practice behaviors.

This study has several limitations. First, the use of convenience sampling may introduce bias. To mitigate this, we took various measures to ensure broad participation in the questionnaire survey and enlarge the sample as much as possible to increase the dependability of the convenience sampling and provide useful information and insights. Second, the use of electronic questionnaires, completed at students’ convenience, may have affected data quality. To address this, we excluded questionnaires with an extremely short completion time or identical answers across all items. Students may also have responded positively rather than reflecting their true situations. To minimize this, we provided clear instructions and emphasized anonymity. The questionnaire was developed and refined by experts, reducing the likelihood of students’ retrieving correct answers. Further, as the study was conducted in seven cities in Southern and Central China, its generalizability may be limited. However, the geographic spread and large sample size offer valuable insights into university students’ KAP regarding antibiotic use in food-producing animals. While the reliability of the questionnaire was not assessed, a Cronbach’s alpha of 0.66, calculated from a random sample of 100 students from this study, indicated acceptable internal consistency and reliability [31]. We recommend future studies to test the reliability and validity of the scale before using it to further confirm the applicability to their targeted population. Future studies could further refine questions related to antibiotic content in food to address potential misunderstandings arising from discrepancies in food labeling regulations across different regions and incorporate educational interventions and health awareness in regression models for more comprehensive findings.

## 4. Materials and Methods

This study applied the STrengthening the Reporting of OBservational studies in Epidemiology (STROBE) statement to guide the report [32]. The STROBE checklist is in Table S1.

### 4.1. Study Design and Setting

This cross-sectional study involved university students from seven cities: five in Yunnan Province (Kunming, Yuxi, Baoshan, Dali, and Lincang City) and two provincial cities of Hu’nan Province (Changsha City) and Henan Province (Zhengzhou City). In Yunnan, the five cities collectively have about 64 universities (52 in Kunming, 3 each in Yuxi and Baoshan, 4 in Dali, and 2 in Lincang), with our study covering 15 universities (respectively 5, 3, 2, 4, and 1). In Changsha and Zhengzhou, which included approximately 60 and 72 universities, we included 20 and 37 universities, respectively. Data were collected between July 2022 to May 2024.

### 4.2. Participants

University students from the seven cities were invited to participate during the data collection period, with no restrictions on gender, age, major, or academic year. Convenience sampling was used to recruit participants.

### 4.3. Measurement

The questionnaire was developed by a clinical epidemiologist (Y.J.H) and a statistician based on previous studies [13,29,33,34,35]. It was piloted on 25 students, revised, and then tested on an additional 100 students to ensure the clarity of the questions. A multidisciplinary team, consisting of epidemiologic and statistic experts (I. X. W, D. Z, and S. D) and an expert in clinical infection (Y. J. H), further refined the questionnaire based on the feedback from the pilot test. Appropriate answers were determined during the development phase. 

### 4.4. Variables

The self-administered questionnaire, consisting of 27 items, was divided into four parts (Table S2): (a) basic information: 12 items covering demographic information, vaccination, and antibiotic use condition; (b) knowledge section: eight items assessing knowledge about antibiotic use in humans and food-producing animals, with response options of “yes”, “no” or “not sure”; (c) attitude section: four items gauging attitudes towards the use of antibiotics for disease treatment, prevention and growth promotion in food-producing animals, with “yes” or “no” responses; (d) practice section: three items measuring attention to antibiotic content in food-producing animals in daily life. Each correct response was awarded one point and separate scores were calculated for each dimension, respectively, with higher scores indicating better levels. 

### 4.5. Study Size

The study was conducted using Wenjuanxing (http://www.wjx.cn/ accessed on 18 July 2022), a widely used online survey platform in China. Participants accessed the questionnaire by scanning a QR code or clicking a link. The required sample size for the study was calculated assuming that 50% [36] of students would demonstrate good KAP scores; using a 95% confidence level (Z = 1.96) and a margin of error of 5%, the initial sample size was determined to be 384 participants using the standard formula for proportions [37]. To account for a 10% non-response rate, the sample size was adjusted to 423 participants. To ensure broad participation, the questionnaire QR code and link were distributed through various channels, including faculty announcements after classes, sharing on university electronic platforms, displaying posters on campus, and inviting students to share with peers. A description of the study and instructions preceded the questionnaire.

### 4.6. Bias

Questionnaires were deemed invalid if (a) completed in less than one minute; (b) the same answer was selected for all the items; or (c) inconsistencies existed among basic information variables, such as age, education, and discipline. Wenjuanxing settings required participants to answer all items, eliminating the issue of missing data. 

### 4.7. Statistical Methods

Data were initially summarized in Microsoft Excel 2016 and analyzed in SPSS version 25.0. Descriptive statistics were applied to summarize basic information and respondence appropriateness rate, presented as mean ± standard deviation or frequencies and percentages. Universities were classified as double-first-class or non-double-first-class universities according to the Ministry of Education of the People’s Republic of China (www.gov.cn accessed on 6 July 2022). Associations between basic information variables and KAP scores were examined using logistic regression, with the KAP considered good if it reached or exceeded the median score [38]. Median scores were used to define good scores, as the median is less influenced by extreme values and better represents the central tendency of the survey data [39]. Covariates were selected based on prior knowledge from literatures. Spearman correlation coefficients were used to analyze the relationships between KAP dimensions and total scores. A significant value level of *p* < 0.05 was considered statistically significant.

## 5. Conclusions

This study found that university students across seven cities in China exhibited low knowledge and poor practices regarding antibiotic use in food-producing animals. Targeted educational interventions should emphasize fundamental concepts and principles of antibiotic use in human and food-producing animals, incorporate antibiotic stewardship messages into vaccination programs, and promote actionable behaviors to combat AMR. Regression analysis suggests these efforts should prioritize younger female students, those in non-medical majors, from non-double-first-class universities, with lower family monthly incomes, and without parents in the medical field, or without recent vaccination history.

## Figures and Tables

**Table 1:** Basic information of participants ^1^.

Characteristic	n (%)/Median (IQR)
**Gender**	
Male	1712 (26.93)
Female	4645 (73.07)
**Age**, years	20.00 (19.00, 21.00)
**Education**	
Undergraduate	5898 (92.78)
Postgraduate	459 (7.22)
**Discipline**	
Natural science	154 (2.42)
Agricultural science	214 (3.37)
Medical science	3790 (59.62)
Engineering and technical sciences	777 (12.22)
Humanities and social sciences	1422 (22.37)
**Double-first-class university** *	
Yes	663 (10.43)
No	5694 (89.57)
**Parents in medical area**	
Yes	1377 (21.66)
No	4980 (78.34)
**Family monthly income**, CNY	
≤3000	2730 (42.95)
3001–10,000	2906 (45.71)
10,001–20,000	537 (8.45)
>20,000	184 (2.89)
**Seasonal influenza vaccination**	
In the past year	1152 (18.12)
In the past two years	1385 (21.79)
Two years before	1891 (29.75)
Never	1929 (30.34)
**COVID-19 vaccination**	
In the past year	1528 (24.03)
In the past two years	3231 (50.83)
Two years before	1516 (23.85)
Never	82 (1.29)
**Antibiotic use in the past year**	
Yes	4095 (64.42)
No	2262 (35.58)

^1^ IQR, interquartile range. * Universities were classified into double-first-class and non-double-first-class universities according to the list launched by the Ministry of Education of the People’s Republic of China.

**Table 2:** Appropriate respondence to knowledge, attitude, and practice items regarding antibiotic use.

Question (Preferred Answer)	Respondence Appropriateness Rate n (%)
**Knowledge**	
Q1. Antibiotics are only effective for treating viral infections (False).	3132 (49.27)
Q2. Antibiotics are only effective for treating bacterial infections (True).	1906 (29.98)
Q3. Antibiotics are effective for managing common cold (False).	1365 (21.47)
Q4. Wider spectrum is better than narrow spectrum antibiotics (False).	1706 (26.84)
Q5. Bacteria which are resistant to antibiotics can be spread from food-producing animals to human (True).	3026 (47.60)
Q6. Antibiotics used in animals can only be prescribed by a veterinarian (True).	2515 (39.56)
Q7. Most of the local food-producing animals are antibiotic free (False).	1420 (22.34)
Q8. Most of the imported food-producing animals are antibiotic free (False).	1552 (24.41)
**Attitude**	
Q1. Do you think we should use antibiotics as disease treatment in food-producing animals? (Yes).	3445 (54.19)
Q2. Do you think we should use antibiotics as prophylactics (disease prevention) in food-producing animals? (No).	3584 (56.38)
Q3. Do you think we should use antibiotics as growth promoters in food-producing animals? (No).	2439 (38.37)
Q4. Do you think farmers should reduce the use of antibiotics in food-producing animals? (Yes).	5217 (82.07)
**Practice**	
Q1. When you buy food from food-producing animals, do you frequently check for antibiotic content in food? (Frequent attention).	1366 (21.49)
Q2. Have you ever refused to purchase food from food-producing animals due to the presence of antibiotics? (Yes).	1812 (28.50)
Q3. How much are you willing to pay for animal meat products without antibiotics? (Higher price than similar food).	3215 (50.57)

**Table 3:** Factors associated with knowledge, attitude, and practice total score based on binary regression analysis ^1^.

Variable	*β*	*p*	OR (95% CI)
**Gender** ^2^			
Female	Ref		
Male	0.117	0.043	**1.124 (1.004, 1.260)**
**Age, year** ^2^	0.156	<0.001	**1.169 (1.135, 1.203)**
**Education** ^3^			
Undergraduate	Ref		
Postgraduate	1.127	<0.001	**3.085 (2.321, 4.101)**
**Discipline** ^4^			
Non-medical science	Ref		
Medical science	0.551	<0.001	**1.736 (1.566, 1.924)**
**Double-first-class university** *^,3^			
No	Ref		
Yes	1.135	<0.001	**3.111 (2.485, 3.893)**
**Family monthly income, CNY** ^2^			
≤3000	Ref		
3001–10,000	0.194	<0.001	**1.214 (1.091, 1.350)**
10,001–20,000	0.541	<0.001	**1.718 (1.408, 2.096)**
>20,000	0.547	0.001	**1.729 (1.249, 2.391)**
**Parents in medical area** ^2^			
No	Ref		
Yes	0.371	<0.001	**1.449 (1.280, 1.642)**
**Seasonal influenza vaccination** ^5^			
Never	Ref		
In the past year	0.261	0.002	**1.298 (1.100, 1.532)**
In the past two years	−0.058	0.441	0.943 (0.813, 1.094)
Two years before	−0.019	0.784	0.981 (0.857, 1.123)
**COVID-19 vaccination** ^5^			
Never	Ref		
In the past year	0.652	0.007	**1.920 (1.195, 3.085)**
In the past two years	0.547	0.021	**1.728 (1.085, 2.752)**
Two years before	0.336	0.162	1.399 (0.874, 2.240)
**Antibiotic use in the past year** ^2^			
No	Ref		
Yes	0.242	<0.001	**1.273 (1.146, 1.414)**

^1^ OR, odds ratio; CI, confidence interval; Ref, reference. ^2^ The model included gender and age. * Universities were classified into double-first-class and non-double-first-class universities according to the list launched by the Ministry of Education of the People’s Republic of China. ^3^ The model included gender, age, and family monthly income. ^4^ The model included gender, family monthly income, and parents in medical area. ^5^ The model included gender, age, education, discipline, double-first-class university, family monthly income, parents in medical area, seasonal influenza vaccination, COVID-19 vaccination, and antibiotic use in the past year.

**Table 4:** Factors associated with knowledge, attitude, and practice score based on binary regression analysis ^1^.

Variable	Knowledge Score			Attitude Score			Practice Score		
	*β*	*p*	OR (95% CI)	*β*	*p*	OR (95% CI)	*β*	*p*	OR (95% CI)
**Gender** ^2^									
Female	Ref			Ref			Ref		
Male	0.079	0.166	1.082 (0.968, 1.210)	−0.038	0.556	0.963 (0.850, 1.091)	0.214	0.001	**1.238 (1.096, 1.398)**
**Age, year** ^2^	0.098	<0.001	**1.103 (1.072, 1.134)**	0.130	<0.001	**1.139 (1.102, 1.177)**	0.094	<0.001	**1.099 (1.066, 1.132)**
**Education** ^3^									
Undergraduate	Ref			Ref			Ref		
Postgraduate	1.305	<0.001	**3.688 (2.835, 4.797)**	1.424	<0.001	**4.1** **53 (2.** **803, 6.** **154)**	−0.195	0.127	0.823 (0.640, 1.057)
**Discipline** ^4^									
Non-medical science	Ref			Ref			Ref		
Medical science	0.735	<0.001	**2.084 (1.881, 2.310)**	0.194	0.001	**1.** **214 (** **1.** **084, 1.** **359)**	−0.172	0.002	**0.842 (0.755, 0.939)**
**Double-first-class university** *^, 3^									
No	Ref			Ref			Ref		
Yes	1.013	<0.001	**2.754 (2.252, 3.368)**	1.979	<0.001	**7.** **233 (** **4.** **958,** **10.552)**	0.232	0.032	**1.261 (1.021, 1.557)**
**Family monthly income, CNY** ^2^									
≤3000	Ref			Ref			Ref		
3001–10,000	0.028	0.601	1.029 (0.926, 1.143)	0.298	<0.001	**1.347 (1.198, 1.515)**	0.272	<0.001	**1.312 (1.174, 1.468)**
10,001–20,000	0.135	0.162	1.145 (0.947, 1.383)	0.652	<0.001	**1.919 (1.516, 2.429)**	0.551	<0.001	**1.736 (1.398, 2.155)**
>20,000	0.396	0.013	**1.485 (1.087, 2.029)**	0.355	0.052	1.426 (0.997, 2.038)	0.303	0.007	**1.354 (0.968, 1.893)**
**Parents in medical area** ^2^									
No	Ref			Ref			Ref		
Yes	0.282	<0.001	**1.326 (1.175, 1.497)**	0.154	0.028	**1.1** **67 (** **1.** **017, 1.339)**	0.488	<0.001	**1.629 (1.420, 1.868)**
**Seasonal influenza vaccination** ^5^									
Never	Ref			Ref			Ref		
In the past year	0.044	0.590	1.045 (0.889, 1.229)	0.015	0.868	1.016 (0.847, 1.218)	0.692	<0.001	**1.997 (1.671, 2.387)**
In the past two years	−0.228	0.002	**0.796 (0.687, 0.922)**	-0.045	0.587	0.956 (0.813, 1.124)	0.419	<0.001	**1.520 (1.302, 1.775)**
Two years before	−0.157	0.021	**0.855 (0.748, 0.977)**	0.006	0.941	1.006 (0.867, 1.166)	0.165	0.017	**1.180 (1.030, 1.351)**
**COVID-19 vaccination** ^5^									
Never	Ref			Ref			Ref		
In the past year	0.587	0.015	**1.798 (1.119, 2.889)**	0.629	0.012	**1.877 (1.150, 3.062)**	−0.113	0.665	0.893 (0.537, 1.487)
In the past two years	0.572	0.016	**1.772 (1.112, 2.824)**	0.494	0.042	**1.639 (1.017, 2.641)**	−0.261	0.306	0.770 (0.468, 1.269)
Two years before	0.542	0.024	**1.719 (1.073, 2.756)**	0.319	0.195	1.375 (0.849, 2.227)	−0.286	0.267	0.751 (0.454, 1.244)
**Antibiotic use in the past year** ^2^									
No	Ref			Ref			Ref		
Yes	0.106	0.045	**1.112 (1.002, 1.234)**	0.244	<0.001	**1.** **276 (1.** **137, 1.** **432)**	0.138	0.014	**1.148 (1.028, 1.282)**

^1^ OR, odds ratio; CI, confidence interval; Ref, reference. ^2^ The model included gender and age; * Universities were classified into double-first-class and non-double-first-class universities ac-cording to the list launched by the Ministry of Education of the People’s Republic of China. ^3^ The model included gender, age, and family monthly income; ^4^ The model included gender, family monthly income, and parents in medical area; ^5^ The model included gender, age, education, discipline, double-first-class university, family monthly income, parents in medical area, seasonal influenza vaccination, COVID-19 vaccination, and antibiotic use in the past year.

**Table 5:** Results of correlation analysis.

Dimension	Knowledge Score	Attitude Score	Practice Score	Total Score
Knowledge score	1.000	-	-	-
Attitude score	0.120 **	1.000	-	-
Practice score	0.065 **	0.082 **	1.000	-
Total score	0.797 **	0.562 **	0.423 **	1.000

^**^ *p* < 0.001.

## Data Availability

The data underlying this article will be shared on reasonable request to the corresponding author.

## Supplementary Materials

The following supporting information can be downloaded at: https://www.mdpi.com/article/10.3390/antibiotics13121189/s1, Table S1: Checklist of STROBE Statement items included in reports of the study; Table S2: Survey on university students’ knowledge, attitude, and practice towards antibiotic use in food-producing animals.
