# Supplementary material for: Knowledge, Attitudes, and Practices Toward Antibiotic Use in Food-Producing Animals Among University Students in Seven Cities in Southern and Central China: A Cross-Sectional Study
Source: Antibiotics (Basel). 2024 Dec 6;13(12):1189. doi: 10.3390/antibiotics13121189 (PMC11672557; doi:10.3390/antibiotics13121189)
Supplement: Supplementary file 1 [file antibiotics-13-01189-s001.zip › antibiotics-3296892-supplementary.pdf]

Table S1. Checklist of STROBE Statement items included in reports of the study

	Item No.	Recommendation	Page No.	Relevant text from manuscript
**Title and abstract**	1	(*a*) Indicate the study's design with a commonly used term in the title or the abstract	1	Knowledge, attitudes, and practices toward antibiotic use in food-producing animals among university students in seven cities in Southern and Central China: A cross-sectional study
		(*b*) Provide in the abstract an informative and balanced summary of what was done and what was found	1	The misuse of antibiotics in both human and food-producing animals poses significant risks to human health and contributes to the rise of antibiotic resistance……These programs should address the fundamental concepts of antibiotic use in both human and food-producing animals, while providing practical guidance on individual behaviors to help mitigate antibiotic resistance.
**Introduction**				
Background/rationale	2	Explain the scientific background and rationale for the investigation being reported	1-2	Inappropriate antibiotic use across human, animal, and environmental sectors is a major driver of antimicrobial resistance (AMR)……One Chinese study on human antibiotics found that students with lower KAP scores were more likely to self-medicate with antibiotics or demand antibiotics from doctors[14].
Objectives	3	State specific objectives, including any prespecified hypotheses	2	To address the gap, a cross-sectional study was conducted across universities in seven cities within Yunnan, Hu'nan, and Henan Provinces, located in Southern and Central China. The study aimed to assess university students' KAP regarding antibiotic use in food-producing animals and to identify potential influencing factors.
**Methods**				
Study design	4	Present key elements of study design early in the paper	9	4.1. Study design and setting<br>This cross-sectional study involved university students from seven cities.
Setting	5	Describe the setting, locations, and relevant dates, including periods of recruitment, exposure, follow-up, and data collection	9-11	4.1. Study design and setting<br>This cross-sectional study involved university students from seven cities: five in Yunnan Province (Kunming, Yuxi, Baoshan, Dali, and

				Lincang City), and two provincial cities of Hu'nan Province (Changsha City) and Henan Province (Zhengzhou City)……A significant value level of $p < 0.05$ was considered statistically significant.
Participants	6	(*a*) *Cohort study*—Give the eligibility criteria, and the sources and methods of selection of participants. Describe methods of follow-up<br>*Case-control study*—Give the eligibility criteria, and the sources and methods of case ascertainment and control selection. Give the rationale for the choice of cases and controls<br>*Cross-sectional study*—Give the eligibility criteria, and the sources and methods of selection of participants	10	4.2. Participants<br>University students from the seven cities were invited to participate during the data collection period, with no restrictions on gender, age, major or academic year. Convenience sampling was used to recruit participants.
		(*b*) *Cohort study*—For matched studies, give matching criteria and number of exposed and unexposed<br>*Case-control study*—For matched studies, give matching criteria and the number of controls per case	Not applicable	Not applicable
Variables	7	Clearly define all outcomes, exposures, predictors, potential confounders, and effect modifiers. Give diagnostic criteria, if applicable	10	4.4. Variables<br>The self-administered questionnaire, consisting of 27 items, was divided into four parts……Each correct response was awarded one point, and separate scores were calculated for knowledge, attitude, and practice respectively, with higher scores indicating better levels.
Data sources/ measurement	8*	For each variable of interest, give sources of data and details of methods of assessment (measurement). Describe	10	4.5. Study size<br>The study was conducted using Wenjuanxing (http://www.wjx.cn/ accessed on 18 July 2022), a widely used online survey platform in

		comparability of assessment methods if there is more than one group		China. Participants accessed the questionnaire by scanning a QR code or clicking a link.
Bias	9	Describe any efforts to address potential sources of bias	10	4.6. Bias<br>Questionnaires were deemed invalid if: a) completed in less than one minute; b) the same answer was selected for all the items; or c) inconsistencies existed among basic information variables, such as age, education, and discipline. Wenjuanxing settings required participants to answer all items, eliminating the issue of missing data.
Study size	10	Explain how the study size was arrived at	10	To ensure broad participation, the questionnaire QR code and link were distributed through various channels, including faculty announcement after classes, sharing on university electronic platforms, displaying posters on campus, and inviting students to share with peers.
Quantitative variables	11	Explain how quantitative variables were handled in the analyses. If applicable, describe which groupings were chosen and why	10	Descriptive statistics were applied to summarize basic information and respondence appropriateness rate, presented as mean ± standard deviation or frequencies and percentages.
Statistical methods	12	(*a*) Describe all statistical methods, including those used to control for confounding	10-11	Descriptive statistics were applied to summarize basic information and respondence appropriateness rate, presented as mean ± standard deviation or frequencies and percentages……Spearman correlation coefficient was used to analyze correlations between KAP dimensions and total scores.
		(*b*) Describe any methods used to examine subgroups and interactions	Not applicable	Not applicable
		(*c*) Explain how missing data were addressed	10	Wenjuanxing settings required participants to answer all items, eliminating the issue of missing data.
		(*d*) *Cohort study*—If applicable, explain how loss to follow-up was addressed	Not applicable	Not applicable

		Case-control study—If applicable, explain how matching of cases and controls was addressed<br>*Cross-sectional study*—If applicable, describe analytical methods taking account of sampling strategy		
		(*e*) Describe any sensitivity analyses	Not applicable	Not applicable
**Results**				
Participants	13*	(a) Report numbers of individuals at each stage of study—eg numbers potentially eligible, examined for eligibility, confirmed eligible, included in the study, completing follow-up, and analysed	2	A total of 7,170 questionnaires were collected, of which 813 were excluded due to either short completion time or inconsistencies in the basic information provided. Finally, 6,357 (88.66%) participants were included in the analysis.
		(b) Give reasons for non-participation at each stage	2	A total of 7,170 questionnaires were collected, of which 813 were excluded due to either short completion time or inconsistencies in the basic information provided.
		(c) Consider use of a flow diagram	Not applicable	Not applicable
Descriptive data	14*	(a) Give characteristics of study participants (eg demographic, clinical, social) and information on exposures and potential confounders	3	Table 1
		(b) Indicate number of participants with missing data for each variable of interest	Not applicable	Not applicable
		(c) *Cohort study*—Summarise follow-up time (eg, average and total amount)	Not applicable	Not applicable
Outcome data	15*	*Cohort study*—Report numbers of outcome events or summary measures over time	Not applicable	Not applicable

		Case-control study—Report numbers in each exposure category, or summary measures of exposure	Not applicable	Not applicable
		Cross-sectional study—Report numbers of outcome events or summary measures	3-7	Table 1-5
Main results	16	(*a*) Give unadjusted estimates and, if applicable, confounder-adjusted estimates and their precision (eg, 95% confidence interval). Make clear which confounders were adjusted for and why they were included	5-7	Table 3-4
		(*b*) Report category boundaries when continuous variables were categorized	3	Table 1
		(*c*) If relevant, consider translating estimates of relative risk into absolute risk for a meaningful time period	Not applicable	Not applicable
Other analyses	17	Report other analyses done—eg analyses of subgroups and interactions, and sensitivity analyses	Not applicable	Not applicable
**Discussion**				
Key results	18	Summarise key results with reference to study objectives	7	This study assessed 6,357 students from seven cities in the Southern and Central China regarding their KAP towards antibiotic use in food-producing animals……Additionally, positive correlations were found between scores of each KAP dimension, as well as the KAP total score.
Limitations	19	Discuss limitations of the study, taking into account sources of potential bias or imprecision. Discuss both direction and magnitude of any potential bias	9	This study has several limitations……Future studies could further refine questions related to antibiotic content in food to address potential misunderstandings arising from discrepancies in food labeling regulations across different regions and incorporate educational interventions and health awareness in regression models for more comprehensive findings.

Interpretation	20	Give a cautious overall interpretation of results considering objectives, limitations, multiplicity of analyses, results from similar studies, and other relevant evidence	8-9	Despite acceptable attitudes, there was still a concerning gap……This finding highlights the need for targeted education programs that consider discipline and factors with contrasting associations, such as education and vaccination history, to improve practice behaviors.
Generalisability	21	Discuss the generalisability (external validity) of the study results	9	Further, as the study was conducted in seven cities in Southern and Central China, its generalizability may be limited. However, the geographic spread and large sample size offer valuable insights into university students' KAP regarding antibiotic use in food-producing animals.
**Other information**				
Funding	22	Give the source of funding and the role of the funders for the present study and, if applicable, for the original study on which the present article is based	11	The research was funded by Natural Science Found of Hunan Province (No. 2023JJ30734).

Table S2. Survey on university students' knowledge, attitude, and practice towards antibiotic use in in food-producing animals

DEMOGRAPHICS

1. What is your sex?
a. Male
b. Female

2. What is your age? ___

3. What is the current degree you are working on?
a. Undergraduate
b. Postgraduate

4. What is/are your major(s)? ___
a. Natural science
a. Agricultural science
b. Medical science
c. Engineering and technical sciences
d. Humanities and social sciences

5. In which university are/were you under? ___

6. When did you take seasonal influenza vaccine:
a. In the past 12 months
b. In the past 24 months
c. Beyond past 24 months
d. Never taken

7. When did you take Covid-19 vaccine:
a. In the past 12 months
b. In the past 24 months
c. Beyond past 24 months
d. Never taken

8. Which of the following best describes your monthly household income, before tax?
Single Code
a. ≤3000 CNY
b. 3001–10,000 CNY
c. 10,001–20,000 CNY
d. >20,000 CNY

9. Is any of your immediate family a medical staff?
a. Yes

b. No

10. Have you used antibiotics in the past 12 months?

a. Yes
b. No

11. if yes to Q10, please indicate where did you get the antibiotics

a. Prescribed by a doctor
b. Bought from a pharmacy store without a prescription
c. My parents gave their leftover
d. My friends gave it to me
e. From my previous stored antibiotics
f. Other (please write down)

12. if yes to Q10, please indicate the reasons for using antibiotics

a cold/running nose or flu like symptom

a. Diarrhea
b. Cough
c. Sore throat
d. Fever
e. Other (please write down)

KNOWLEDGE (yes/no/I don't know)

1. Antibiotics are only effective for treating viral infections.
2. Antibiotics are only effective for treating bacterial infections.
3. Antibiotics are effective for managing common cold
4. Wider spectrum is better than narrow spectrum antibiotics
5. Bacteria which are resistant to antibiotics can be spread from food-producing animal to human
6. Antibiotics used in animals can only be prescribed by a veterinarian
7. Most of the local food-producing animals are antibiotic free
8. Most of the imported food-producing animals are antibiotic free

ATTITUDE

9. Do you think we should use antibiotics as disease treatment in food-producing animals?

a. Yes
b. No

10. Do you think we should use antibiotics as prophylactics (disease prevention) in food-producing animals?

a. Yes
b. No

11. Do you think we should use antibiotics as growth promoter in food-producing animals?

a. Yes
b. No

12. Do you think farmers should reduce the use of antibiotics in food-producing animals?
a. Yes
b. No

PRACTICE

13. When you buy food from food-producing animals, do you frequently check for antibiotic content in food?
a. Frequently
b. Occasionally
c. Never (skip to question 15)

14. Have you ever refused to purchase food from food-producing animals due to the presence of antibiotics?
a. Yes
b. No

15. How much are you willing to pay for animal meat products without antibiotics?
d. Price of similar food
a. Higher than 0%-25% price of the similar food
b. Higher than 26%-50% price of the similar food
c. Higher than 51%-75% price of the similar food
d. Higher than 76%-100% price of the similar food
